# Scientometric analysis of lipid metabolism in breast neoplasm: 2012–2021

**DOI:** 10.3389/fphys.2023.1042603

**Published:** 2023-04-25

**Authors:** Xiaobing Lin, Qiuping Yang, Daitian Zheng, Huiting Tian, Lingzhi Chen, Jinyao Wu, Zeqi Ji, Yexi Chen, Zhiyang Li

**Affiliations:** Department of Thyroid, Breast and Hernia Surgery, General Surgery, The Second Affiliated Hospital of Shantou University Medical College, Shantou, Guangdong, China

**Keywords:** breast neoplasm, lipid metabolism, scientometrics, bibliometrix, VOSviewer, CiteSpace

## Abstract

**Introduction:** In recent years, more and more studies have proved that lipid metabolism plays an essential role in breast cancer’s proliferation and metastasisand also has a specific significance in predicting survival.

**Methods:** This paper collected data from 725 publications related to lipid metabolism in breast neoplasm from 2012 to 2021 through the Web of Science Core Collection database. Bibliometrix, VOSviewer, and CiteSpace were used for the scientometrics analysis of countries, institutions, journals, authors, keywords, etc.

**Results:** The number of documents published showed an increasing trend, with an average annual growth rate of 14.49%. The United States was the most productive country (*n* = 223, 30.76%). The journals with the largest number of publications are mostly from developed countries. Except for the retrieved topics, “lipid metabolism” (*n* = 272) and “breast cancer” (*n* = 175), the keywords that appeared most frequently were “expression” (*n* = 151), “fatty-acid synthase” (*n* = 78), “growth” (*n* = 72), “metabolism” (*n* = 67) and “cells“ (*n* = 66).

**Discussion:** These findings and summaries help reveal the current research status and clarify the hot spots in this field.

## 1 Introduction

Female breast neoplasm overtook lung cancer and became the most common cancer in 2020 (Sung et al., 2021). Moreover, breast neoplasm was women’s number one cancer killer (Sung et al., 2021). With high morbidity and mortality, breast cancer poses a significant threat to human life, health, and safety. The existing methods of breast cancer treatment, like chemotherapy and radiation therapy, have various adverse reactions, affecting the prognosis of patients. Therefore, it is particularly significant to explore prognostic risks while advancing the research of therapeutic interventions (Wang and Wang, 2022).

As crucial biomolecules, lipids are not only the component of cell membranes but also signal transduction molecules and energy sources (Bian et al., 2021; Ward et al., 2021; Batchuluun et al., 2022). An increasing number of studies have shown that lipid metabolism is abnormal in cancer (Faubert et al., 2020; Snaebjornsson et al., 2020; Bian et al., 2021). A variety of cancers can have a high metabolic rate through lipids (Snaebjornsson et al., 2020), which are involved in tumorigenesis and metastasis (Bian et al., 2021). What is noteworthy is that breast cancer is closely related to lipid metabolism. Breast cancer is mainly divided into hormone receptor (HR) positive subtype, human epidermal growth factor 2 (HER2) positive subtype, and triple-negative subtype, showing different degrees of lipid dependence (Ward et al., 2021). It has been identified that APOL4, NR1H3, and other genes related to lipid metabolism are independent prognostic markers of breast neoplasm and can predict overall survival in breast cancer patients (Chang and Xing, 2022; Wang and Wang, 2022). Lipid metabolism is likely to be a breakthrough for targeted therapy of breast cancer (Ward et al., 2021). The progress of lipidomics has strengthened the research on lipids of breast cancer, but metabolic drugs for breast cancer treatment are still undeveloped and need to be further studied (Ward et al., 2021). In conclusion, the study of lipid metabolism in breast cancer has significance in the treatment and prediction of survival.

Scientometrics uses mathematical and statistical methods to analyze knowledge carriers, among which the Web of Science is the most commonly used database (Chen et al., 2019; Dong et al., 2019; Perazzo et al., 2019; Huang et al., 2021). However, the scientometric analysis of lipid metabolism in breast cancer has not yet been conducted. Based on this premise, this paper conducted a scientometric analysis of literature from 2012 to 2021, included in the Web of Science Core Collection in this field, to clarify the research trend and to lay the foundation for future research. This paper aims to clarify the most published countries and journals as well as the most influential authors, articles, and keywords in the past decade. Finally, our study summarized the development trend of lipid metabolism in breast cancer and provided a possible direction for future development.

## 2 Materials and methods

**FIGURE 1:**
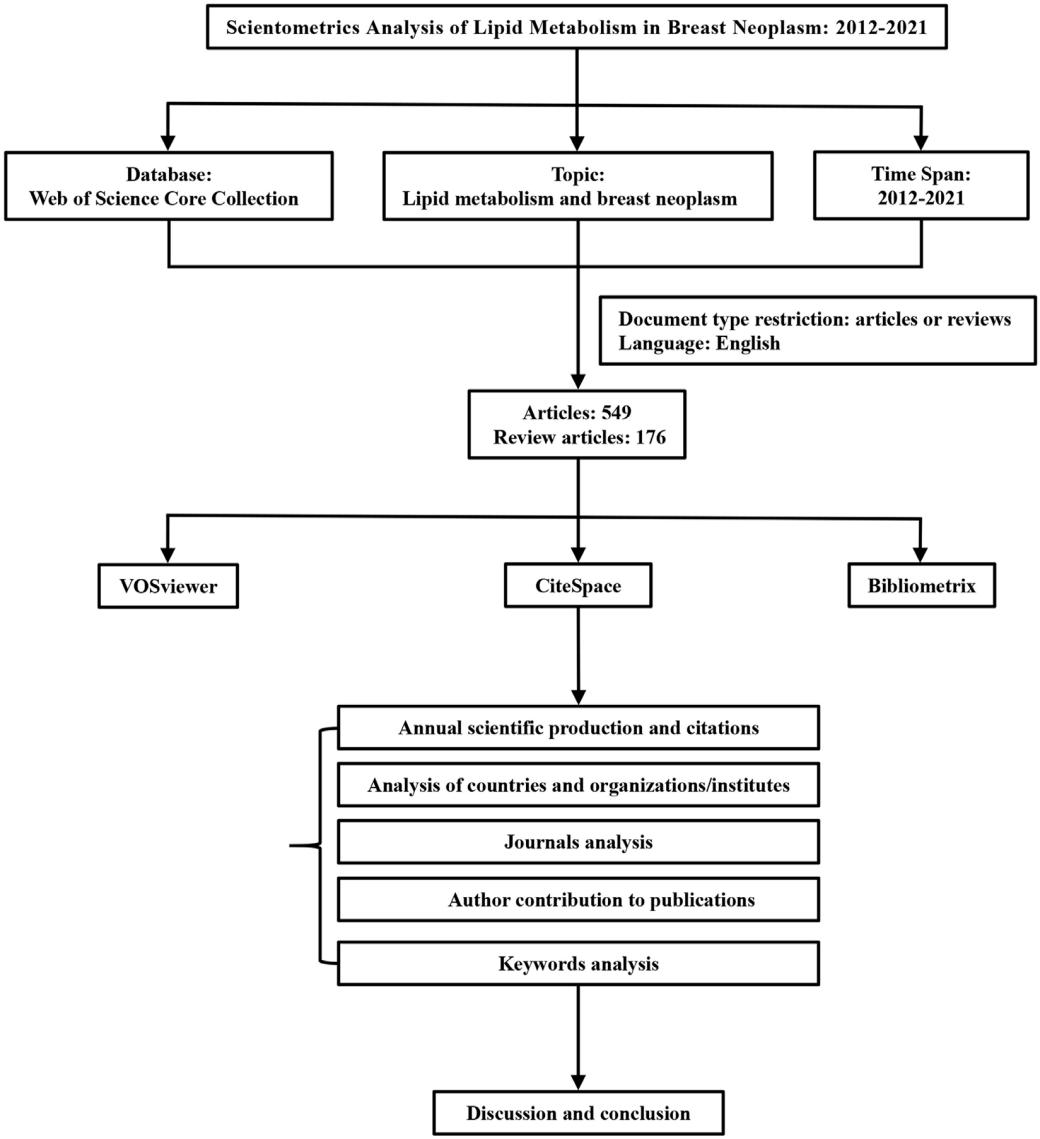
Flow chart of the scientometric analysis.

Web of Science Core Collection database was used to collect information, and scientometric analysis was conducted by bibliometrix, CiteSpace, and VOSviewer. The specific process is shown in Figure 1.

### 2.1 Data collection

Web of Science Core Collection database includes literature from databases including SCI-EXPANDED (2003-present), SSCI (2003-present), A&HCI (2003-present), ESCI (2015-present), CCR-EXPANDED (1985-present), and IC (1993-present) (Chen et al., 2022). When carrying out visual analysis through CiteSpace, using WoS may achieve a better effect (Falagas et al., 2008; Martín-Martín et al., 2018). Therefore, this paper selected the Web of Science Core Collection database for retrieving and collecting data for scientometric analysis.

By looking up the Medical Subject Heading, we got the subject terms “breast neoplasm”, “lipid metabolism” and their synonyms, and then searched in the Web of Science Core collection database. The specific search formula is as follows: #1, TS= (“lipid metabolism”); #2, TS= (“Breast Neoplasm*”) OR TS= (“Breast Tumor*”) OR TS= (“Breast Cancer*”) OR TS= (“Mammary Cancer*”) OR TS= (“Breast Carcinoma*”) OR TS= (“Mammary Carcinoma*”) OR TS= (“Mammary Neoplasm*”); #3, “#1” AND “#2”. The literature retrieval was implemented on 26 August 2022. Before refining, the number of results obtained was 1048. A total of 752 articles, 267 review articles, 22 meeting abstracts, 16 proceeding papers, seven book chapters, six early access, six editorial material, 1 data paper, one letter, and one retracted publication were retrieved. After that, the time horizon was limited to 2012–2021, and the language was restricted to English. The document types we chose were “article” and “review article”, with 557 (75.58%) and 180 (24.42%) papers, respectively. In fact, by using Web of Science’s “marked list”, “refine results”, and other features, we’ve found that 12 of the articles were published in 2022, so we excluded them. Altogether, 725 documents containing 549 articles (75.72%) and 176 review articles (24.28%) were involved in the analysis. All of these records were exported in plain text, including information such as author, title, publication title, language, document type, abstract, source, PubMed ID, and so on.

### 2.2 Data analysis

In addition to data collection, the Web of Science can also be used for preliminary data analysis. For example, getting a citation report to obtain information about citing articles, citing articles without self-citations, citing times, citing times without self-citations, H-Index, G-index, M-index, the highest cited publication, and the like. The H-index is a measure of academic achievement. H stands for “high citations”. A researcher’s H-index is defined as having at most H papers cited at least H times each. The higher a person’s H-index, the more influential his/her papers. Considering that the H-index has shortcomings, such as the impact of highly cited papers cannot be well reflected, the G-index is added to supplement it. G-index is the derivative index of the H-index, which is defined as: in descending order of citations, such that the sum of the citations of all articles in the top G is greater than the square of G, and the maximum value that can meet the conditions is the value of G (Ali, 2021). Given the feature that H-index is beneficial to scholars who have been publishing for years, M-index normalizes H-index by considering the number of years of publication (Hirsch, 2005). All of the information obtained, including the author, organization, keywords, and so forth, was imported into the relevant software for analysis and graph drawing. The specific software is described as follows.

VOSviewer (version 1.6.18, Leiden, Netherlands) is a program that can be used to construct or view bibliometric maps (Ma et al., 2021). We can analyze co-authorship, co-occurrence, citation, bibliographic coupling, and co-citation through the program. After setting a certain value, the qualified data can be screened to make visual pictures. In this study, VOSviewer was used to analyze the citation of publications, co-citation references, countries, and keywords.

Bibliometrix is an R-tool of R-studio (version 4.2.1, Naples, Italy). Biblioshny is a web interface for Bibliometrix (Campra et al., 2022), which can be used to make network maps and show trends. It was applied to analyze the annual production and average citations per year, the most cited documents and countries, the most productive institutions, journals and authors, and keywords in this text.

CiteSpace is a scientometric analysis software (Chen, 2004) which can realize data visualization (Zhu et al., 2020). The software includes a variety of functions, such as network diagram generation, cluster analysis, burst keyword finding, and so on. The version of this software is constantly updated. The version used in this article is CiteSpace, V.6.1.R2 (Philadelphia, United States). In this study, we used CiteSpace to analyze countries and keywords (“burst words” and clustering analysis).

## 3 Results

### 3.1 Annual scientific production and citations

**FIGURE 2:**
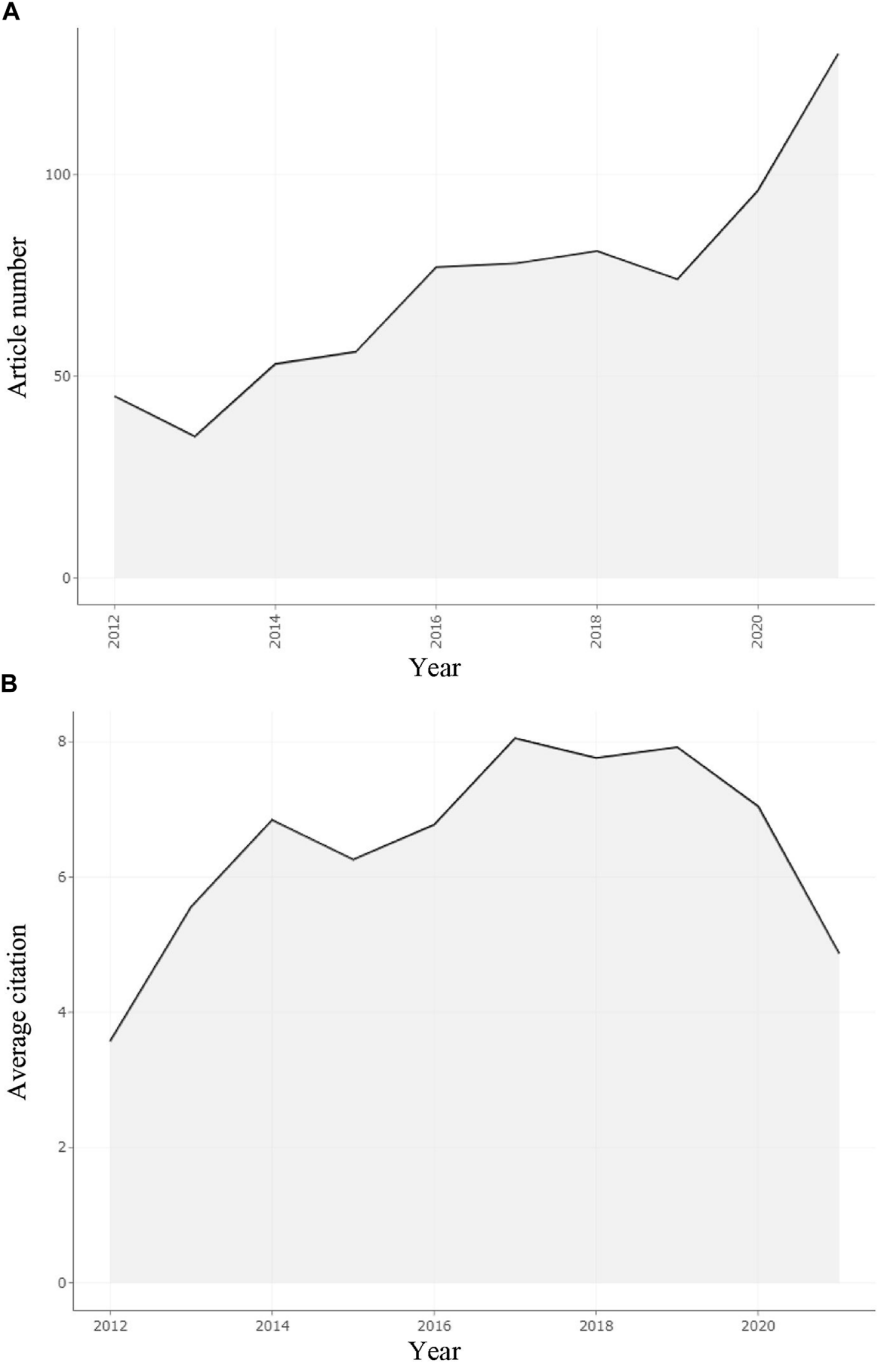
Annual scientific production and average citations per year from 2012 to 2021. **(A)** Annual scientific production from 2012 to 2021 in the field. **(B)** Average article citations per year from 2012 to 2021 in the field.

From 2012 to 2021, the Web of Science Core Collection database included 725 publications (articles and review articles) on lipid metabolism in breast neoplasm. The trend of the annual quantity of published articles is shown in Figure 2A. On the whole, the number of publications was on an upward trend. The number of publications increased from 45 in 2012 to 130 in 2021, with an average annual growth rate of 12.51%. 2021 was the year with the largest increase in publications, while 2014 had the highest growth rate of 51.43 percent. It was worth noting that the peak occurred in 2021, and the number of publications was 130, accounting for 17.93% of the total. However, there was a slight decrease in 2013 and 2019, with 35 (4.83%) and 74 (10.21%) publications, respectively. In 2016 (77, 10.62%), 2017 (78, 10.76%), and 2018 (81, 11.17%), the number of essays was almost equal.

In the decade from 2012 to 2021, a total of 725 publications in this field have been cited 21237 times, including 20415 non-self-citations, with a 29.29 average per item. “Non-self-citations” is the total number of citations except the author’s own. “Average per item” means the average citations of these papers. It refers to the total number of citations divided by the number of publications. The H-index was 69, which means that 69 papers had been cited at least 69 times.Figure 2B shows the average citations per year. Apart from the last 2 years, the number of citations, on the whole, was on the rise. There were slight declines in 2015 (to 6.26), 2018 (to 7.76), and 2020 (to 7.04). The most notable decline was in 2021, when the average annual citation was only 4.87. The highest average citation per year was in 2017 (8.05). The fastest increase in the number of articles was in 2013.

**FIGURE 3:**
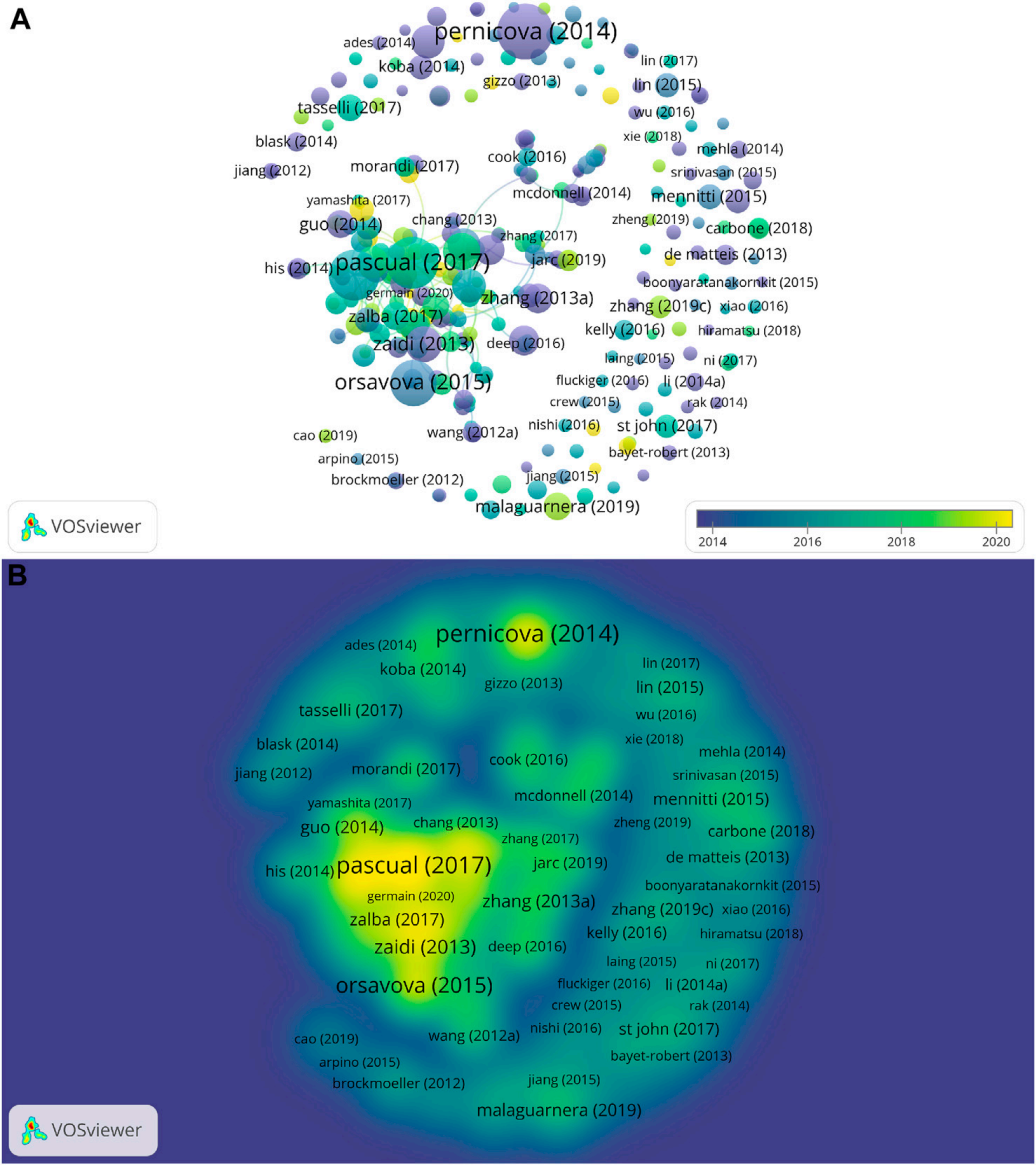
Overlay map and density map of documents with more than 20 citations. **(A)** Overlay map of documents with more than 20 citations. **(B)** Density map of documents with more than 20 citations.

Among all the articles published in this field from 2012 to 2021, three articles (0.41%) were cited more than 500 times. Ten articles were cited more than 200 times and occupied 1.38% of all documents, and 32 articles (4.41%) were cited over 100 times. The top ten cited papers can be seen in Table 1. The most cited document (Pernicova et al., 2014) was “Metformin-mode of Action and Clinical Implications for Diabetes and Cancer”, with a total of 712 times (Pernicova and Korbonits, 2014). This paper is a review published in *Nature Reviews Endocrinology*. In second place was “Targeting metastasis-initiating cells through the fatty acid receptor CD36” by Pascual et al., with 607 citations (Pascual et al., 2017). It is an article published in *Nature*. The third cited article was “The multifaceted roles of fatty acid synthesis in cancer” (Röhrig et al., 2016), which was cited 581 times in total (Röhrig and Schulze, 2016). It is also a review that came out in *Nature reviews Cancer*. The fourth-most cited article (Orsavova et al., 2015) was cited 473 times, while the No.5 cited article (Wang et al., 2018) had 295 citations. Figure 3 shows overlay map and density map of documents. To display the publications as much as possible and clearly, we set the publications to be cited over 20 times. Each ball in Figure 3A represents a document in this field. The size of the ball indicates the number of citations. The larger the ball, the more times it was cited. The color of the ball indicates the year. The closer to purple indicates an earlier publication and the closer to yellow indicates a newer publication. In Figure 3A, it is clear that the most cited document (Pernicova et al., 2014) was published in 2014, which is earlier, so the ball is purple. What is more, the lines between balls mean their relationship. The relationship between these publications about lipid metabolism in breast neoplasm was not close. Density visualization is shown in Figure 3B. The more points with greater weight around a node, the closer the node’s color is to yellow.

**TABLE 1:** Top 10 cited documents in the field.

R	Author	Title	Source	TC	TC/Y
1	Pernicova and Korbonits (2014)	Metformin-mode of action and clinical implications for diabetes and cancer	Nature reviews Endocrinology	712	79.11
2	Pascual et al. (2017)	Targeting metastasis-initiating cells through the fatty acid receptor CD36	Nature	607	101.17
3	Röhrig and Schulze (2016)	The multifaceted roles of fatty acid synthesis in cancer	Nature reviews Cancer	581	83.00
4	Orsavova et al. (2015)	Fatty Acids Composition of Vegetable Oils and Its Contribution to Dietary Energy Intake and Dependence of Cardiovascular Mortality on Dietary Intake of Fatty Acids	International journal of molecular sciences	473	59.13
5	Wang et al. (2018)	JAK/STAT3-Regulated Fatty Acid β-Oxidation Is Critical for Breast Cancer Stem Cell Self-Renewal and Chemoresistance	Cell metabolism	295	59.00
6	Zaidi et al. (2013)	Lipogenesis and lipolysis: the pathways exploited by the cancer cells to acquire fatty acids	Progress in lipid research	282	28.20
7	Camarda et al. (2016)	Inhibition of fatty acid oxidation as a therapy for MYC-overexpressing triple-negative breast cancer	Nature medicine	245	35.00
8	Römpp et al. (2013)	Mass spectrometry imaging with high resolution in mass and space	Histochemistry and cell biology	239	23.90
9	Lochner et al. (2015)	Fatty acid metabolism in the regulation of T cell function	Trends in immunology	226	28.25
10	Cheng et al. (2018)	Lipid metabolism reprogramming and its potential targets in cancer	Cancer communications	221	44.20

Notes: R = rank, TC, total citations, TC/Y = total citations per year.

**FIGURE 4:**
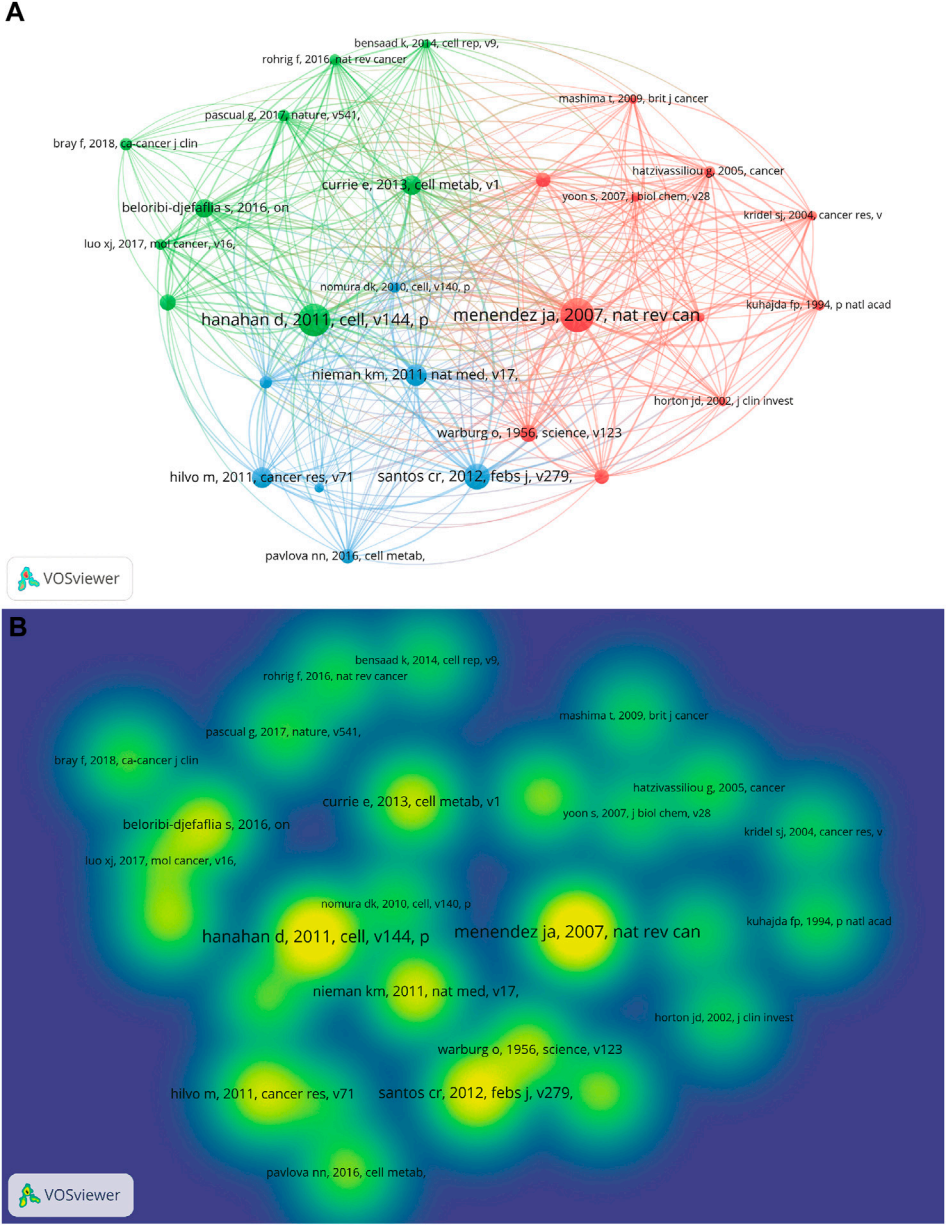
Co-citation network map and density map of cited references. **(A)** Co-citation network map of cited references of all the publications in this field. **(B)** Co-citation density map of cited references of all the publications in this field.

Similarly, the co-cited references of all the publications in this field have been visualized (Figure 4A; Figure 4B). Co-cited literature refers to one or more articles cited by one or more articles (Xu et al., 2021). To clearly show the information of the co-cited references, we set the minimum number of citations of a cited reference as 24, and 27 (0.06%) met the threshold. The references were grouped into three clusters and presented in three different colors (red, green, and blue). The most cited reference (Menendez JA et al., 2007) was cited 96 times (Menendez and Lupu, 2007). The second (Hanhan D et al., 2011) and third (Santos cr et al., 2012) references were cited 91 and 71 times (Hanahan and Weinberg, 2011; Santos and Schulze, 2012), respectively, by these publications. The density view is shown in Figure 4B.

### 3.2 Analysis of countries and organizations/institutes

**FIGURE 5:**
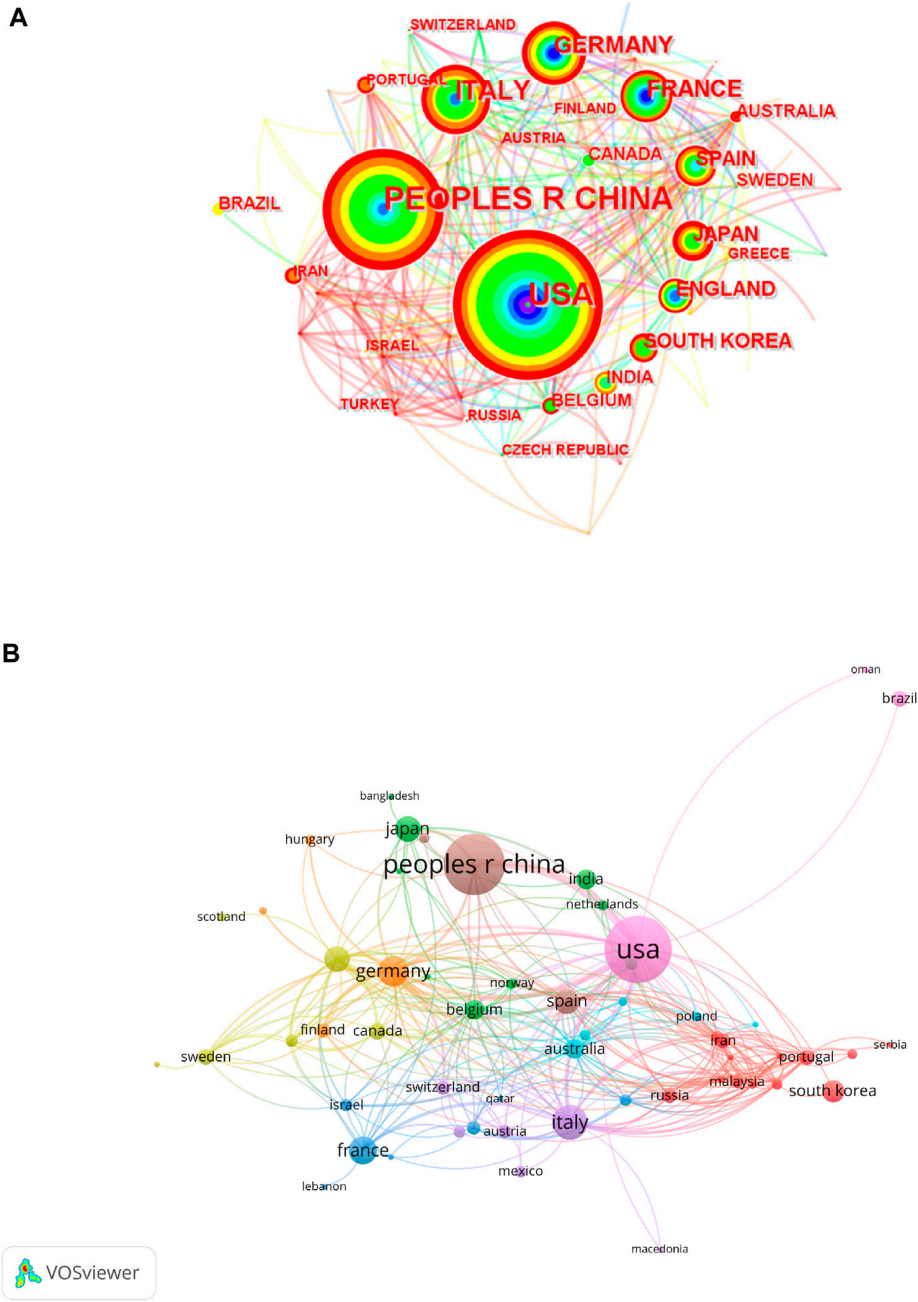
Production and cooperation map of the country. **(A)** Network map of national publication volume and cooperation between countries. **(B)** Co-authorship network map of countries.

The number of publications issued by countries and the cooperation between them can be seen in Figure 5. In Figure 5A, each circle represents a node. The larger the circle is, the more documents the country contributed. The red circle in the outermost indicates that the country has recently published articles. The larger the circle of a particular color, the more articles were published in that year. For Figure 5B from VOSviewer, every circle also represents a country. The larger circle reflects the country’s larger number of publications. What is different is that the line between nodes not only represents the connection between them but also reflects the distance between nodes, which indicates the close degree of connection.

Figure 5A shows that out of all the 63 countries, the United States was the most productive country, with 223 publications, counting for 30.76% of the total. China ranked second with 184 documents (25.38%). These two countries led the rest of the world in terms of output. It was worth noting that these two countries have still had relatively high numbers of publications in recent years. Italy came in third with 61, Germany fourth with 46, and France ranked fifth with 40. Of the 20 most published countries or regions, 14 are developed countries. In terms of geographical location, 10 (50%) of the top 20 published countries are from Europe, 6 (30%) are from Asia, 2 (10%) are from North America, 1 (5%) is from South America and 1 (5%) is from Oceania. The United States also had the highest total link strength, which was 147 (Figure 5B). The other top five countries were Germany (60), Italy (58), China (57), and England (Thomas et al., 2019). As for citations, as the country with the largest number of publications, the United States was also the country that had been cited most frequently (Table 2). The citation frequency was 7767. Similarly, China, as the second most published country, also ranked second (*n* = 3735) in the number of citations. Not surprisingly, the vast majority of the top ten countries are developed countries, with nine, while China is the only developing country.

**TABLE 2:** Top 10 most cited countries.

Country	Total citations
United States	7767
China	3735
Germany	2143
Italy	2007
United Kingdom	1829
Spain	1490
France	1256
Belgium	801
Japan	739
Czech Republic	654

From the perspective of institutions (Table 3), among the 1,316 organizations, the University of Texas MD Anderson Cancer Center had the largest number of articles, with 13 articles or 3.17 percent of the total documents. Shanghai Jiao Tong University followed with 10, counting for 1.38%. Zhejiang University also published 10 (1.38%) documents. Harvard Medical School, Sun Yat-Sen University, and Central South University ranked fourth to sixth, with 9 documents (1.24%). Of the top 10 institutions, 8 are located in China, and 2 are in the United States.

**TABLE 3:** Top 10 institutions with the largest number of publications.

Affiliation	Articles
University of Texas MD Anderson Cancer Center	13
Zhejiang University	10
Shanghai Jiao Tong University	10
Sun Yat-Sen University	9
Harvard Medical School	9
Central South University	9
Sichuan University	8
Peking University	8
Fudan University	8
Chinese Academy of Sciences	8

### 3.3 Journals analysis

Seven hundred twenty-five articles on lipid metabolism in breast cancer were published in 354 journals. Among them, *PLoS One* published the most articles, with 25 articles, accounting for 3.45% of the total documents, followed by the *International Journal of Molecular Sciences* (*n* = 20, 2.76) and *Scientific Reports* (*n* = 20, 2.76%) with the same number of essays. *Oncotarget* (*n* = 17, 2.34%) ranked third place. Of the top ten journals, 4 are from England, 3 from Switzerland, 2 from the United States, and 1 from the Netherlands. It can be seen in Table 4 that *PLoS One*, with the most significant number of publications, also had the highest H-index (Ali, 2021), G-index (Orsavova et al., 2015), and M-index (1.455). Expect for *Oncotarget*, the impact factors for these journals ranged from 3.752 (*PLoS One*) to 13.312 (*Cancer Research*). In addition, among the top 10 journals in terms of the number of publications, four other journals had an H-index of 10 or greater, namely *Oncotarget* (Chen et al., 2022), *Scientific Reports* (Perazzo et al., 2019), *Cancer Research* (Perazzo et al., 2019), and *International Journal of Molecular Sciences* (Chen et al., 2019). However, the most globally cited journal was the *International Journal of Molecular Sciences* (*n* = 906), ranked fifth in the number of publications. The journals with the highest volume of publications were overwhelmingly cancer-related. Half of the journals: *Oncotarget*, *Cancer Research*, *Frontiers in Oncology*, *BMC Cancer*, and *Cancers*, mainly include articles related to cancer, which were classified as “oncology”. One of these journals (*Nutrients*) was devoted to nutrition, which may be associated with the topic of this paper, “lipid metabolism”. Others were listed as “multidisciplinary sciences” or “biochemistry & molecular biology”.

**TABLE 4:** Top 10 journals for research of lipid metabolism in breast neoplasm.

Element	H-index	G-index	M-Index	TC	NP	PY-start	IF 2022	Q
PLoS One	16	25	1.455	750	25	2012	3.752	Q2
Oncotarget	13	17	1.444	462	17	2014	/	/
Scientific Reports	12	20	1.500	459	20	2015	4.996	Q2
Cancer Research	12	14	1.091	534	14	2012	13.312	Q1
International Journal of Molecular Sciences	10	20	1.250	906	20	2015	6.208	Q1,Q2
Frontiers in Oncology	8	14	1.143	258	14	2016	5.738	Q2
Biochimica Et Biophysica Acta-Molecular and Cell Biology of Lipids	7	9	0.636	435	9	2012	5.228	Q1,Q2
BMC Cancer	7	8	0.875	319	8	2015	4.638	Q2
Cancers	5	11	1.250	141	13	2019	6.575	Q1
Nutrients	4	9	1.000	177	9	2019	6.706	Q1

Notes: TC, total citation; NP, number of publications; PY-start = publication year start; IF, impact factor; Q = quartile in category.

### 3.4 Author contribution to publications

In total, 4,804 authors contributed to the documents about lipid metabolism in breast neoplasm. Five authors published single-author articles. The international co-authorship rate was 27.03%, and the average number of authors per article was 7.59. The top ten authors’ publications and impacts are shown in Table 5. Wang J and Li J were the authors with the most significant publications, with 9 articles. Li L, Li Y, Wang Y, and Zhao Y, who published 8 articles respectively, followed closely. Twenty-five authors published more than or equal to 5 articles. The author with the most significant number of publications, Li J, had published relevant articles since 2014 and continued to post articles until 2021. Another author who also published the most articles, Wang J, started publishing articles about related fields as early as 2012. Among the top ten authors by the number of articles, Wang J and Swinnen JV were the first to publish relevant articles.

**TABLE 5:** Publication and impact of the top ten authors by the number of articles.

Authors	Articles	H-index	G-index	M-Index	TC	PY-start
Li J	9	7	8	0.778	170	2014
Wang J	9	7	9	0.636	235	2012
Li L	8	5	8	0.556	129	2014
Li Y	8	4	7	0.500	240	2015
Wang Y	8	7	8	0.700	187	2013
Zhao Y	8	8	8	0.889	149	2014
Chen J	7	3	6	0.429	57	2016
Swinnen JV	6	5	6	0.455	494	2012
Yang X	6	5	6	0.556	117	2014
Yang Y	6	3	6	0.429	51	2016

Notes: TC, total citation; PY-start = publication year start.

In terms of citations, however, the most cited author was not the one who published the most articles. Korbonits M topped the list with 712 citations. Pernicova I was also cited 712 times. Schulze A came in third with 645 citations. And then, 13 authors had 607 citations. A total of 17 authors had been cited more than 500 times. The number of authors cited more than 300 times was 31. Seventy-one authors were cited more than 200 times. Zhao Y had the highest H-index, which was 8. Li J, Wang J, and Wang Y all had an index of 7. The author with the highest G-index was Wang J. Four authors, Zhao Y, Li J, Wang Y, and Li L, were next, all with a G-index of 8.

### 3.5 Keywords analysis

**FIGURE 6:**
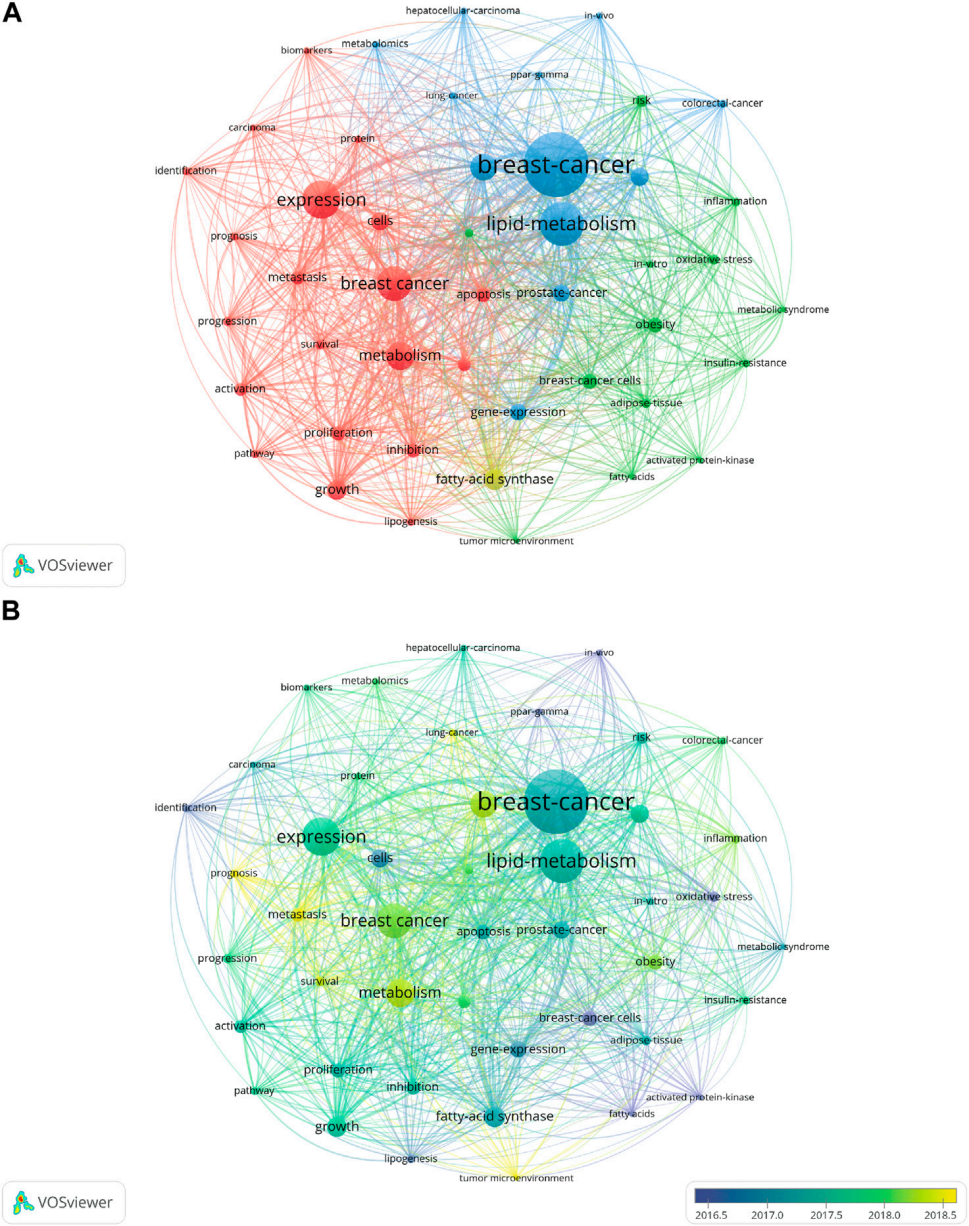
Co-occurrence network map and density map of keywords with more than 20 occurrences. **(A)** Co-occurrence network map of keywords with more than 20 occurrences. **(B)** Overlay visualization map of keywords with more than 20 occurrences.

**FIGURE 7:**
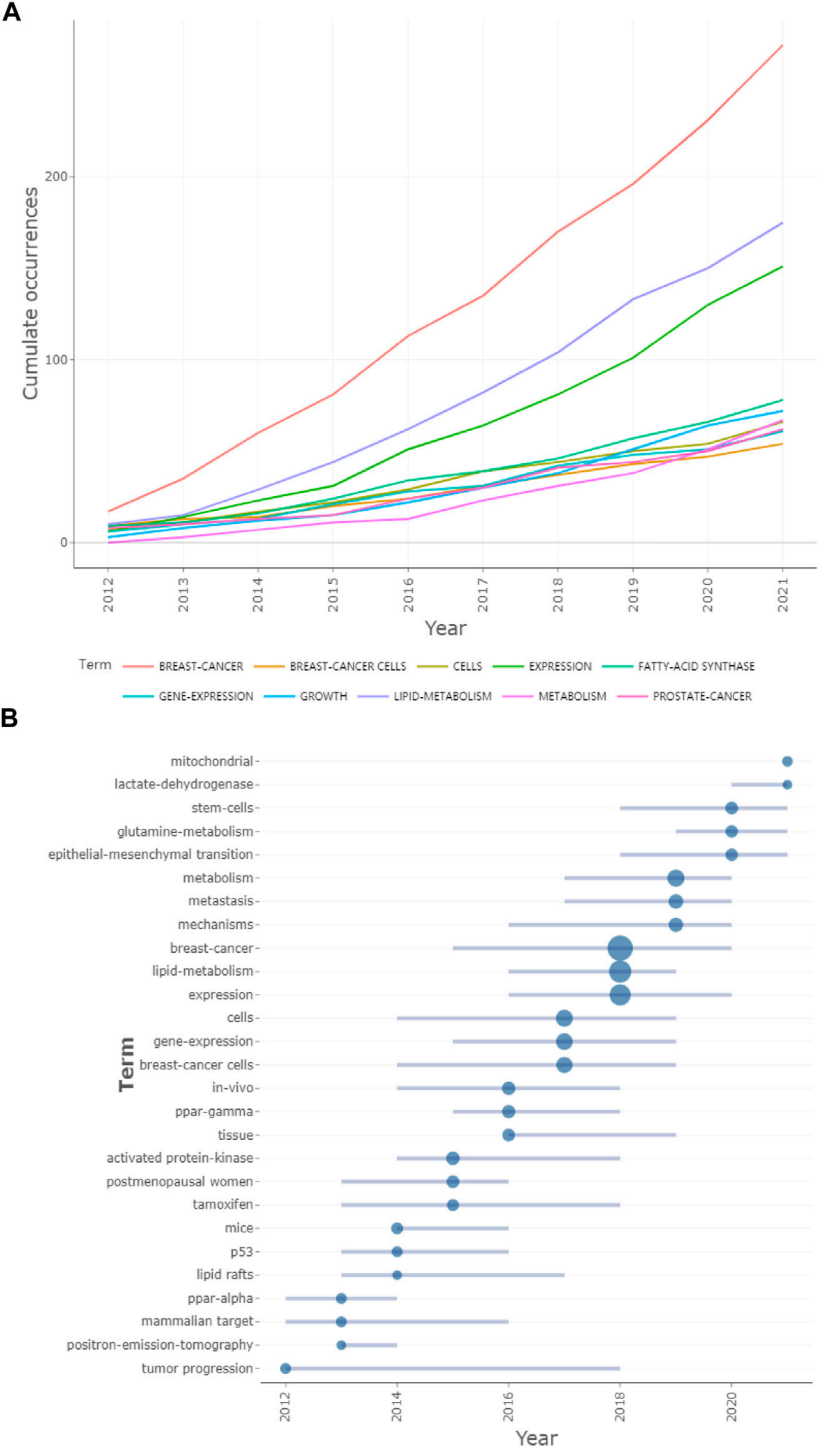
The trend of the top 10 keywords in frequency and trend topic. **(A)** The trend of the top ten most frequently used keywords. **(B)** Trend topic of lipid metabolism in breast neoplasm.

Among all the 3909 keywords, “breast-cancer” (272 times) showed the highest number of occurrences, followed by “lipid-metabolism” (175 times), which fit the theme. In addition to these two words, the other top 10 keywords were “expression” (151 times), “breast cancer” (136 times), “metabolism” (108 times), “lipid metabolism” (102 times), “fatty-acid synthase” (78 times), “growth” (73 times), “cancer” (67 times), and “cells” (66 times). Forty-six keywords met the condition of appearing more than 20 times, which could be divided into four major clusters and shown in four colors: red, yellow, green, and blue (Figure 6A). The overlay visualization map is shown in Figure 6B. “Expression” had the most significant increase except for “breast-cancer” and “lipid-metabolism”, from 6 in 2012 to 151 in 2021 (Figure 7A). The popularity of keywords varied from year to year (Figure 7B). Between 2012 and 2013, the keywords “tumor progression”, “positron-emission-tomography”, “mammalian target”, and “ppar-alpha” were more popular, while around 2019, “metabolism”, “metastasis” and “mechanisms” were significantly more frequently used. Especially around 2020, the three keywords “stem-cells”, “glutamine-metabolism”, and “epithelial-mesenchymal transition” have attracted more attention. According to CiteSpace, all of these keywords could be divided into 10 clusters. They were blood glucose, fatty acid synthesis, fatty acid, nf kappa, metabolic reprogramming, ppar gamma, peroxisome proliferator-activated receptor, profiling lipid distribution, metabolomic profile, and downstream signal. From 2012 to 2016, there was a general trend, starting with the study of tumor development (“tumor progression”) and its diagnosis technology (“positron-emission-tomography”), moving to the molecular (“lipid rafts”, “activated protein-kinase”) and gene level (“ppar-alpha”, “p53”, “ppar-gamma”). In addition, animals (“mammalian target”, “mice”) and live experiments (“*in-vivo*”) also occupied a certain proportion in this stage of research. In 2016, the keyword “lipid-metabolism” began to gain people’s attention. Furthermore, in 2018, “lipid-metabolism” appeared with considerable frequency (175). Since then, the keywords about metabolism (“metabolism” and “glutamine-metabolism”) had constantly emerged and kept this up until 2020. And then, we used CiteSpace’s “burstiness” function to get 10 burst keywords. According to the year of the outbreak from far to near, the order was “activated protein kinase”, “ppar alpha”, “arachidonic acid”, “postmenopausal women”, “ovarian cancer”, “*in vivo*”, “nuclear receptor”, “abnormal lipid metabolism”, “insulin resistance” and “prognosis”. Hot words of the last 2 years were “insulin resistance”, “resistance”, “prognosis”, and “promote”.

## 4 Discussion

By collecting data from the Web of Science Core Collection and then using Bibliometrix, VOSviewer, and CiteSpace, a scientometrics analysis was implied based on documents on “lipid metabolism in breast neoplasm” from 2012 to 2021.

From 2012 to 2021, there were 725 documents in this field published, and the number of publications showed an increasing trend. While only 45 articles were published in 2012, the number has grown to 130 in 2021, with an average annual growth rate of 12.51 percent. And in line with that, the peak of the number of published articles appeared in 2021, indicating that the number of published articles may continue to show a growing trend in the future. 2016 is the year with a relatively fast growth rate in the number of articles published. Coincidentally, since 2016, keywords related to lipid metabolism have sprung up like mushrooms. It must be noted that in 2015, the WoS introduced ESCI (Emerging Sources Citation Index), adding 7800 titles to the Core Collection, which may be why 2016 was the year with the highest annual growth rate. All in all, it can not be denied that the research on lipid metabolism in breast cancer has gradually come into people’s view and played an increasingly important role in scientific research.

These articles have been cited 21237 times, with an average of 29.29 citations. Except for 2020 and 2021, the number of citations showed an increasing trend. Noteworthily, the number of citations in 2021 was nearly three times that of 2012, indicating that articles in this field play a more significant role in scientific research. The H-index was 69. The H-index, which considers the number of articles and the number of citations (Ciriminna and Pagliaro, 2013; Dinis-Oliveira, 2019), is one of the most commonly used tools for assessing scientific productivity (Díaz et al., 2016).

From the perspective of a single document, the one with the most significant number of citations was “Metformin-mode of Action and Clinical Implications for Diabetes and Cancer” (Pernicova and Korbonits, 2014), with 712 citations. This document was published in *Nature Review Endocrinology* in 2014 with an impact factor of 47.564. Metformin can activate 5’ -AMP-activated protein kinase (AMPK) (Hawley et al., 2002; Gelling et al., 2003; Miller et al., 2013), thereby affecting lipid metabolism. Moreover, metformin has been proven to have anti-mammary tumor effects *in vivo* and *in vitro* (Pernicova and Korbonits, 2014). In estrogen/progesterone receptor positive human breast cancer cell lines (MCF7) and estrogen receptor/progesterone receptor/human epidermal growth factor receptor 2 negative cell lines (MDA-MB-231), metformin has an inhibitory effect on mTORC1, which is an essential switch of tumor growth (Jahani and Davoodi, 2022). A cohort study has shown that metformin use reduces the risk of luminal A breast cancer (estrogen/progesterone receptor positive, human epidermal growth factor receptor 2 negative) in postmenopausal patients with type 2 diabetes (Chikermane et al., 2022). There seems to be an inseparable relationship between metformin, lipid metabolism, and breast cancer. In primary breast cancer, metformin can upregulate multiple genes regulating fatty acid oxidation (FAO), and induce a notable reduction of FAO, leading to lipid accumulation (Lord et al., 2020). An *in vitro* experiment showed that metformin-induced triglyceride fatty acids (TGFA) accumulation in 2 cell lines: estrogen/progesterone receptor positive human breast cancer cell lines (MCF7) and estrogen/progesterone receptor negative cell lines (MDA-MB-468), which was not affected by siRNA-mediated AMPK silencing (Lord et al., 2020). As the transcription of the FAO genes is positively related to the expression of proliferation signature, metformin may play a role in primary breast cancer therapy. Interestingly, disruption of FAO promotes a more epithelial cell phenotype, blocking epithelial-to-mesenchymal transition and reversing the tumorigenicity and drug resistance of basal-like breast cancer (Loo et al., 2021). However, there is no mention of a direct relationship and mechanism between FAO, metformin, and breast cancer. Thus, the therapeutic effect of metformin on luminal A breast cancer and basal-like breast cancer and its mechanism deserve further study. The role of FAO in this process also deserves further discussion. The other two top 3 highly cited articles mainly introduced the correlation between metabolism and cancer. Fatty acid receptor CD36 is associated with the prognosis and metastasis of Luminal A breast cancer. When CD36 is deficient, Luminal A breast cancer metastasis is inhibited, and the survival rate is high, suggesting that it is relevant to prognosis (Pascual et al., 2017). Various tumor processes, such as structural synthesis, depend on lipid synthesis, and some lipid-metabolizing enzymes may be potential targets for cancer therapy (Röhrig and Schulze, 2016).

The 725 articles published between 2012 and 2021 involved 63 countries. These countries are spread across different continents and include both developed and developing countries. Seven over ten of the top 20 countries are developed countries, and only 30 percent are developing countries. It can be a reason that developed country amply supported with funds so that they have enough money to invest in scientific research. Although the increase in the burden of breast cancer is more pronounced in low-developing countries (Li et al., 2019), the high incidence and mortality of breast cancer should not be taken lightly by any country. China, a developing country, came in second among the top five countries. It may be due to the development of China’s economy. Moreover, the incidence and mortality of breast cancer in China are not to be overlooked. In 2020, China ranked 65th in age-standardized incidence and 13th in age-standardized mortality, higher than the global average (Cao et al., 2021). From the perspective of geographical location, the countries with a large number of publications are primarily distributed in Europe. In contrast, the others are scattered on other continents, which may also be related to the fact that most developed countries are concentrated in Europe. As for the number of citations, the United States, as the most prolific country, was also the most cited country, with 7,767 citations. Nine of the top ten most cited countries are developed countries, which reflects their contribution to this field of research. Countries with more publications and citations also had a higher degree of cooperation with other countries.

In terms of institutions, a total of 1316 institutions participated in the publication of the article, among which eight of the top ten institutions are from China. Therefore, the investment and contribution of developing countries in this field of research could not be denied.

From 2012 to 2021, 354 journals published articles on lipid metabolism in breast cancer.*PLoS One* was the journal with the most publications, with 25 documents. However, the journal with the largest number of citations (*International Journal of Molecular Sciences* with 906 citations) was not the same one with the most significant number of publications. It is worth mentioning that most of the top 10 journals are related to cancer. Most journals were classified as “oncology” or “biochemistry & molecular biology”. As it turned out, researchers focused on lipid metabolism and breast cancer at the molecular mechanism level. It may be a choice for researchers in this field to consider these journals when submitting materials. Although journals with a large number of publications are not concentrated in one or two countries, most of them are distributed in developed countries, again confirming the advanced nature and contribution of developed countries in this field.

For the 725 articles, a total of 4,804 authors participated and contributed. The average number of authors per article was 7.59, and the international co-authorship rate was 27.03%. Two authors published the most articles, Li J and Wang J, with 9 papers each. The most cited author was not the one with the most articles, but Korbonits M and Pernicova I, with 712 citations. However, they do not rank in the top 10 regarding the number of posts, which may also be why their H-index is not so high. There were 14 authors whose H-index is greater than or equal to 5. The number of authors with an H-index greater than 5 is 4, they were Zhao Y, Li J, Wang J, and Wang Y, indicating that these 4 authors have made outstanding contributions to this field and are essential scientific researchers.

Keywords are the embodiment of the critical points of the article (Liu et al., 2012). The scientometric analysis of keywords can reveal the key issues and development trends of the research field (Li et al., 2016). From 2012 to 2021, a total of 3909 keywords were used in articles related to this field. The two most frequently appeared were “breast-cancer” and “lipid-metabolism”, with 272 and 175 occurrences, respectively, which is consistent with the theme. The other top 10 keywords were “expression”, “breast cancer”, “metabolism”, “lipid metabolism”, “fatty-acid synthase”, “growth”, “cancer”, and “cells”. The usage of CiteSpace can get “burst words”. “Burst words” refer to words that are frequently used over a while and can help researchers predict trends in a research field (Luo et al., 2021). The latest hot terms were “insulin resistance”, “resistance”, “prognosis”, and “promote”, which began in 2018 and continued until 2020 or 2021, suggesting that these four keywords may be hot words for future research. Hyperinsulinism, as an early marker of metabolic dysfunction, is considered to be an independent risk factor and prognostic factor for breast cancer (Senapati et al., 2019; Thomas et al., 2019). Notably, metformin, which was mentioned in the most cited literature, can increase insulin sensitivity of peripheral tissues, especially muscle tissues, and achieve a hypoglycemic effect (Pernicova and Korbonits, 2014). Metformin plays a role in increasing energy stress in cancer cells (Pernicova and Korbonits, 2014). The anti-cancer effects of metformin have also been shown in breast cancer (Pollak, 2013). More interestingly, metformin inhibits breast cancer growth and migration induced by hyperinsulinemic stimulation (Scordamaglia et al., 2022). This suggests that inhibition of hyperinsulinism may be another mechanism of metformin against breast cancer and again demonstrates the potential therapeutic value of metformin in breast cancer. In addition, “prognosis” is likely to become one of the hot spots in recent years. More and more researchers have used lipid metabolism-associated genes (LMAGs) or RNA data to establish models and successfully predict the prognosis of breast cancer (Gong et al., 2021; Ye et al., 2021; Shi et al., 2022). DYNLT1, IMMT, RAB2A, and SLC25A5 are independent prognostic factors for invasive breast carcinoma and confer a poor prognosis 2). FABP7 and NDUFAB1 also have prognostic value. It is worth noting that FABP7 expression is upregulated in triple-negative breast cancer (Kwong et al., 2019). FABP7 is required for HER2-positive breast cancer cell growth by up-regulating key metastatic genes and pathways (Cordero et al., 2019). FABP7 may serve as a therapeutic target for HER2-positive breast cancer or other types. The study of genes related to lipid metabolism is conducive to the development of targeted drugs and breast cancer prognosis. Since “gene expression” and “prognosis” were essential keywords these years, this topic may still be a hot point for research.

Moreover, we have also explored the Research Fronts Clarivate Analytics reports for our analyzed period in medicine. The series of reports cluster to reveal the annual hot frontiers and emerging frontiers in basic science through big data and bibliometric analysis methods. According to the reports, the hot and emerging field of clinical medicine is inseparable from cancer every year. Although they are primarily about other tumors, such as prostate cancer, these reports highlight the importance and attention of targeted drug therapy and immunotherapy for tumors. The same is true for breast cancer, which is why we are trying to propose new ideas in the field of breast cancer research.

There are still some limitations in our study. The data source is the Web of Science Core Collection database. Although it is a good choice for scientometric analysis, it also has shortcomings. For example, it contains a limited number of journals and does not include all literature in the field. However, WoS has been almost the optimal choice for scientometric analysis. Our study only screened English literature, which may cause certain limitations. The country of the journal is not directly displayed, but we can search for it with the help of the National Library of Medicine. In addition, in the author analysis, the abbreviation of the author’s name cannot be distinguished, so we may be unable to avoid some errors. Our study analyzed various kinds of data, such as countries, authors, and keywords. It proposed possible trends which may have a certain significance for future research ideas in this field.

## 5 Conclusion

In recent years, more and more attention has been paid to lipid metabolism in breast cancer. Lipid metabolism plays a significant role in regulating energy acquisition, growth, and development of breast cancer cells and is related to prognosis. This paper analyzes articles related to lipid metabolism in breast cancer and reveals the contribution and cooperation of countries, institutions, journals, and authors. We also propose the hot fields’ changes and possible development trends by analyzing important literature and keywords. “Epithelial-mesenchymal transition”, “Glutamine-metabolism”, “stem-cells”, “lactate-dehydrogenase”, and “mitochondrial” are emerging fields in recent years. In the future, conducting more in-depth research on these fields may be possible. “Insulin resistance” and “prognosis” are the latest burst words, which may remain the hot topic in the next period.

## Data Availability

The original contributions presented in the study are included in the article/Supplementary Material, further inquiries can be directed to the corresponding authors.

## Abbreviations

HR, hormone receptor; HER2, human epidermal growth factor 2; APOL4, apolipoprotein L4; NR1H3, nuclear receptor subfamily 1 group H member 3; SCI-EXPANDED, Science Citation Index Expanded; SSCI, Social Sciences Citation Index; A&HCI, Arts&Humanities Citation Index; ESCI, Emerging Sources Citation Index; CCR-EXPANDED, Current Chemical Reactions; IC, Index Chemicus; WoS, Web of Science; TC, total citations; NP, number of publications; PY-start, publication year start; LMAGs, lipid metabolism-associated genes; FAO, fatty acid oxidation; TGFA, triglyceride fatty acids.
